# Prevalence of nevi, atypical nevi, and lentigines in relation to tobacco smoking

**DOI:** 10.1371/journal.pone.0254772

**Published:** 2021-07-20

**Authors:** Birgit Sadoghi, Karin Schmid-Zalaudek, Iris Zalaudek, Regina Fink-Puches, Anna Niederkorn, Ingrid Wolf, Peter Rohrer, Erika Richtig

**Affiliations:** 1 Department of Dermatology and Venerology, Medical University of Graz, Graz, Austria; 2 Division of Physiology, Otto-Loewi Research Center for Vascular Biology, Immunology and Inflammation, Medical University of Graz, Graz, Austria; IPATIMUP/i3S, PORTUGAL

## Abstract

**Background:**

Melanocytic nevi have a complex evolution influenced by several endogenous and exogenous factors and are known risk factors for malignant melanoma. Interestingly, tobacco use seems to be inversely associated with melanoma risk. However, the association between tobacco use and nevi and lentigines has not yet been evaluated.

**Methods:**

We investigated the prevalence of nevi, atypical nevi, and lentigines in relation to tobacco smoking in a cohort of 59 smokers and 60 age- and sex-matched nonsmokers, using a questionnaire and performing a total body skin examination by experts.

**Results:**

No significant differences were detected between smokers and nonsmokers in the numbers of nevi, atypical nevi, and lentigines in sun-exposed areas (p = 0.966, 0.326, and 0.241, respectively) and in non-sun-exposed areas (p = 0.095, 0.351, and 0.546, respectively).

**Conclusion:**

Our results revealed no significant differences in the prevalence of nevi, atypical nevi, and lentigines between smokers and nonsmokers in sun-exposed and non-sun-exposed areas.

## Introduction

The development of melanocytic nevi is complex and is influenced by constitutional and environmental factors. In addition to exposure to ultraviolet (UV) radiation, transient or chronic immunosuppression has been linked with eruptive nevi. This suggests a possible positive proliferative effect of reduced immune surveillance on melanocytic proliferation and the development of nevi.

Tobacco contains a wide range of carcinogenic substances and exerts toxic and immunosuppressive effects on several organs, including the skin [1, 2]. Several studies have investigated the association between smoking and skin cancer. A clear correlation was found between smoking and cutaneous squamous cell cancer [1, 2] and, to a lesser extent, basal cell carcinoma [1–3], while current studies report an inverse correlation between smoking and melanoma [1, 2, 4, 5].

There is evidence that a large number of nevi are one of the strongest risk factors for melanoma. However, besides the reported “protective” effect of smoking on the development of melanoma, to our knowledge, no study to date has investigated the potential relationship between smoking and the numbers and clinical appearance of melanocytic nevi and lentigines. The aim of this study was to investigate the correlations between nevi, atypical nevi, and lentigines and tobacco smoking.

## Materials and methods

### Study design and patients

Participants were recruited during the Styrian public skin cancer prevention program”sun.watch.,” supported by Austrian Cancer Aid/Styria, which took place in public swimming baths between June and August 2009. Bathers were informed by leaflets and study personnel at the entrance about the possibility of participating in a skin cancer prevention program and its location. In addition, regular loudspeaker announcements were made during the day. Persons interested in participating came to the reception tent and were informed anonymously by study personnel about the purpose and design of the study. Participation in the study was voluntary and all participants or, in the case of children, their legal representatives gave oral informed consent before enrollment.

All participants agreeing to take part in the study were asked to complete an anonymous questionnaire with information about their sex, age, history of skin cancer and sunburn, and smoking habits, followed by a personal interview and total body skin examination performed by experienced dermatologists. All this information was collected from the participants as part of the above-mentioned public health program for skin cancer. The results of this study are based on the responses to the questionnaire.

### Questionnaire

Questionnaire items concerning smoking behavior included age at onset of smoking, number of cigarettes smoked per day, number of smoking years, and number of years off smoking. “Smokers” were defined as persons who were “currently smoking, or had formerly smoked, at least 20 cigarettes per day for a period of at least 15 consecutive years.” Taking at least 15 years of smoking into account, the age range of study subjects evaluated for our hypothesis was limited to 30–75 years. Nonsmokers were included if they had never smoked before.

A supplementary questionnaire was completed for each participant by the medical staff who performed skin cancer screening (dermatologists who were either experienced residents or board-certified dermatologists). The questionnaire included information about skin type and the numbers of total nevi, atypical nevi, and lentigines. Skin type was assessed as type I to III according to Fitzpatrick [6]. The criteria for atypical nevus were defined according to Garbe et al. [7] as nevus with at least three of the following features: diameter ≥ 5 mm, ill-defined border, irregular margin, varying color within the lesion, and papular and macular components. In addition to the general clinical inspection, dermoscopy was used at the discretion of the examiner. If pathological findings were detected, the participants were instructed to present themselves to a dermatologist for further diagnostic or therapeutic procedures.

### Screening of buttock and shoulder

During the total skin examination, the numbers of nevi, atypical nevi, and lentigines on the left shoulder and left buttock were counted with the use of a 10 × 10-cm template that was applied to the body site. The left buttock was defined as a non-sun-exposed area and the left shoulder as a sun-exposed area. Before the screening, the dermatologists and residents were trained in standardizing the procedure and counting the lesions to minimalize biases from different examiners.

### Statistical analysis

Statistical analysis was performed by SPSS Vers. 19.0 for windows (Statistical Package for the Social Sciences, Chicago, IL, USA). The significance of age differences between the groups (smokers vs. nonsmokers) was determined by the independent-samples *t*-test. Because of the non-normal distribution of skin lesion counts, nonparametric statistics (the Mann-Whitney U test) were used for the analysis of nevi, atypical nevi, and lentigines. The frequency of nevus counts was analyzed by the χ^2^ test. For all tests, a p value < 0.05 was considered to indicate a statistically significant difference.

## Results

### Study group

From a total of 627 volunteers participating in this medical prevention program, a subsample was extracted for inclusion in the present study, consisting of 119 white participants (51 females and 68 males). The smokers group consisted of 20 current smokers and 39 former smokers, for a total of 59 tobacco-consuming participants. There were 60 nonsmokers. The two groups were matched by age and sex.

### Age and sex of participants

The mean age of the participants was 45 years for current smokers, 53 years for former smokers, and 50 years for nonsmokers (Table 1). Smokers and nonsmokers did not differ significantly in age (t_(117)_ = 0.54, p = 0.96). There were more males than females in the smokers group (40 vs. 19) and slightly more females than males in the nonsmokers group (32 vs. 28). There were no significant differences between females and males in the prevalence of any skin lesion.

**Table 1:** Age of participants (years).

Variable	Current smokers	Former smokers	Nonsmokers
N	20	39	60
Mean	44.65	52.69	50.08
Standard deviation	10.65	11.17	12.05
Minimum	30	31	31
Maximum	71	75	72

### Current smokers and former smokers: Smokers group

Smokers (n = 20) started smoking between the ages of 14 and 20 years (mean, 16.60 years), smoked an average of 23.00 cigarettes per day (range, 20–30), and smoked for a mean of 28.05 years (range, 16–54 years). Former smokers (n = 39) started smoking between the ages of 12 and 18 years (mean, 15.87 years), smoked an average of 28.33 cigarettes per day (range, 20–100), and smoked for a mean of 24.95 years (range, 15–50 years). Details of the questionnaire are given in Table 2.

**Table 2:** Smoking behavior of participants.

Variable	Smokers (n = 20)	Former smokers (n = 39)
Mean age at onset of smoking (years)	16.60	15.87
Standard deviation	2.09	1.47
Minimum	14	12
Maximum	20	18
N	20	39
Mean number of cigarettes per day	23.00	28.33
Standard deviation	3.77	13.69
Minimum	20	20
Maximum	30	100
N	20	39
Mean years smoked	28.05	24.95
Standard deviation	9.94	8.79
Minimum	16	15
Maximum	54	50

### Dermatological examination

The most frequent skin type in both groups was type II. Although there were more cases of type III among smokers than among nonsmokers, the difference was not significant (χ^2^ = 2.436, p = 0.296). There were no significant differences between smokers and nonsmokers in the number of actinic lentigines or actinic keratoses (χ^2^ = 0.865, p = 0.649; χ^2^ = 0.123, p = 0.726, respectively). There was also no significant difference between smokers and nonsmokers in the number of cases of chronic light-induced skin damage (χ^2^ = 0.845, p = 0.358). The total numbers of nevi and atypical nevi, as estimated by experts, were classified as none, 1–10, 11–20, 21–50, and 51–100 (for the total number of nevi only). There were no significant differences between smokers and nonsmokers in the distribution of the total number of nevi (χ^2^ = 5.553, p = 0.325) or the number of atypical nevi (χ^2^ = 4.730, p = 0.316). The results of the dermatological examinations are given in Table 3.

**Table 3:** Results of dermatological examination.

	Smokers (n = 59)	Nonsmokers (n = 60)	
What skin type has the participant?			p = 0.296
Type I	7 (12%)	9 (15%)	
Type II	26 (44%)	34 (57%)	
Type III	24 (41%)	17 (28%)	
Type IV	0	0	
Missing information	2 (3%)	0	
**How many actinic lentigines are present in this participant?**			p = 0.649
None	18	19	
Some (< 10)	15	20	
Many (≥ 10)	24	21	
**Are there actinic keratoses present in this participant?**			p = 0.726
Yes	5 (8%)	6 (10%)	
No	50 (85%)	48 (80%)	
Missing information	4 (7%)	6 (10%)	
**Does the participant have chronic light-induced skin damage?**			p = 0.358
Yes	15 (25%)	18 (30%)	
No	37 (63%)	30 (50%)	
Missing information	7 (12%)	12 (20%)	
**Total number of nevi**			p = 0.325
0	1 (2%)	0 (0%)	
1–10	18 (30.5%)	20 (33.3%)	
11–20	18 (30.5%)	11 (18.3%)	
21–50	14 (24%)	24 (40%)	
51–100	3 (5%)	3 (5%)	
Missing information	5 (8%)	2 (3.3%)	
**Number of atypical nevi**			p = 0.316
0	36 (61%)	37 (62%)	
1–10	15 (25%)	17 (28%)	
11–20	4 (7%)	2 (3%)	
21–50	0 (0%)	3 (5%)	
Missing information	4 (7%)	1 (2%)	

### Participants’ history

Twenty-six participants (nonsmokers and smokers) reported having had at least one prior medical examination of their nevi, before this campaign. There was no significant difference between nonsmokers and smokers in the percentage of participants who recognized any change in their nevi within the past year, although more smokers (32.8%) than nonsmokers (13.8%) reported having had up to 11–20 sunburns in their life (χ^2^ = 9.718, p = 0.045). There was no significant difference between nonsmokers and smokers in the self-reported number of sunburns during childhood (χ^2^ = 5.843, p = 0.119) (Table 4).

**Table 4:** Participants’ history.

	Smokers (n = 59)	Nonsmokers (n = 60)	
**Did you recognize any change in your nevi within the past year (including changes in color or size)?**			**p = 0.333**
No	37 (68.5%)	45 (83.4%)	
Yes	17 (31.5%)	9 (16.7%)	
Missing information	5	6	
**How many prior sunburns have you had during your life?**			**p = 0.045**
I had no prior sunburn ever	0 (0.0%)	4 (6.9%)	
1–5	14 (24.1%)	20 (34.5%)	
6–10	15 (25.9%)	17 (29.3%)	
11–20	19 (32.8%)	8 (13.8%)	
>20	10 (17.2%)	9 (15.5%)	
Missing information	1	2	
**How many prior sunburns did you have during childhood?**			**p = 0.119**
None	5	14	
Rarely	35	26	
Frequent	8	6	
Not sure	10	10	
Missing information	1	4	
**Have you had skin cancer?**			
Yes	2	3	

### Nevi and lentigines in the template on the left shoulder

The mean number of nevi on the left shoulder estimated with the use of a 10 × 10-cm template was 1.79 in nonsmokers and 1.84 in smokers, with no significant difference between the groups (p = 0.966). The difference between smokers and nonsmokers in the number of atypical nevi was also not significant (p = 0.326). The mean number of lentigines was 15.43 in nonsmokers and 18.95 in smokers, with no significant difference between the groups (p = 0.241) (Table 5).

**Table 5:** Mean numbers of nevi and lentigines on the shoulder and buttock.

Type of skin lesion and template location	Nonsmokers (n = 59)	Smokers (n = 60)	p
Number of nevi	1.79	1.84	0.966
Template left shoulder 10 × 10 cm			
Number of atypical nevi	0.02	0.00	0.326
Template left shoulder 10 × 10 cm			
Number of lentigines	15.43	18.95	0.241
Template left shoulder 10 × 10 cm			
Number of nevi	0.42	1.09	0.095
Template left buttock 10 × 10 cm			
Number of atypical nevi	0.02	0.00	0.351
Template left buttock 10 × 10 cm			
Number of lentigines	0.59	1.26	0.546
Template left buttock 10 × 10 cm			

## Nevi and lentigines in the template on the left buttock

The mean number of nevi on the left buttock estimated with the use of a 10 × 10 cm template was 0.42 in nonsmokers and 1.09 in smokers. Although there were more nevi in the group of smokers, the difference was not significant (p = 0.095). There were also no significant differences between smokers and nonsmokers in the numbers of atypical nevi and lentigines (p = 0.351 and 0.546, respectively). There were no atypical nevi in smokers (Table 5).

### Age dependency

Stratification of the results by age revealed no significant differences between nonsmokers and smokers aged 30–40 years in the numbers of nevi, atypical nevi, and lentigines on the left shoulder or buttock. Among participants aged 41–50 years, the number of lentigines on the left shoulder was significantly greater in smokers (mean, 23.79) than in nonsmokers (mean, 11.21; t_(36)_ = −2.413, p = 0.021), but no other differences were found. Similarly, among participants aged 51–60 years and greater than 60 years, there were no differences between nonsmokers and smokers in the numbers of nevi, atypical nevi, and lentigines on the shoulder or buttock.

## Discussion

It is well-documented that the number of nevi is a risk factor for the development of melanoma. The risk of melanoma is increased by 4- to 5-fold by a total number of nevi above 50 and by 8- to 10-fold by a total number of nevi above 100 [7–9]. Factors that are also associated with increased numbers of nevi are extensive sun exposure during childhood, intermittent exposure to high-intensity UV radiation, sunburn, fair skin, ephelides, genetic factors, and mutations (e.g., MAP kinase activation) [8].

Tobacco and UV radiation have detrimental effects on the skin [10, 11]. Smoking decreases cutaneous blood flow and depresses immune responses [12]. Smoking is strongly associated with impaired wound healing, wrinkled skin, skin aging, squamous cell carcinoma, psoriasis, and acne inversa [1, 13].

Remarkably, although tobacco use is associated with a number of cancers, studies investigating the relationship between tobacco use and melanoma have reported an inverse association [1, 2, 4, 5, 12]. However, in patients aged 60 or older, environmental factors seem to play a much greater role, since the combination of smoking and sun exposure represents a serious risk factor for the development of melanoma [14], and use of tobacco seems to be an independent risk factor for melanoma [14].

The results of our study suggest that there is no evidence for an association between the occurrence of nevi and the use of tobacco. There were no differences between smokers and nonsmokers in the templates in sun-exposed areas or non-sun-exposed areas with respect to the numbers of total nevi, atypical nevi, and lentigines.

One possible explanation for this finding might be immune depression by smoking, possibly protecting melanocytes from inflammatory processes induced by UV radiation, as suggested by Odenbro et al. [12]. That study investigated a potential relationship between invasive cutaneous melanoma and melanoma in situ with respect to tobacco smoking in Swedish male construction workers and found a decreased risk of cutaneous melanoma and melanoma in situ in tobacco users.

Another explanation could be a decrease in tissue oxygen caused by cigarette smoking, inducing vasoconstrictive effects, arterial hypoxia, or interference with oxygen transport [15]. The skin is a main target of oxidative stress from reactive oxygen species, as described by Trouba et al. [16]. Reactive oxygen species are part of normal cellular processes. However, elevated levels can contribute to cutaneous disorders, even skin cancer [16]. Because melanin synthesis is oxygen dependent [17], this might be another factor for reduced nevogenesis. Furthermore, Pavel et al. showed that increased phaeomelanogenesis in dysplastic nevi was connected with oxidative imbalance, as shown by increased intracellular concentrations of reactive oxygen species [18].

Another explanation for the lack of correlation between smoking and the prevalence of nevi and lentigines could be that smokers spend more time indoors because of their reduced physical activity [12].

Vitamin D might also play a role, since smokers have a higher risk of developing vitamin D deficiency [19]. The major source of vitamin D is cutaneous synthesis by UV radiation. Vitamin D deficiency is involved in several biological processes, including the skin immune system. Vitamin D_3_ affects the proliferation, differentiation, and apoptosis of cells, but its function is dependent on the presence of its receptor. Brozyna et al. investigated the expression of vitamin D receptor in various skin lesions and found the strongest expression in normal skin, followed by skin with melanocytic nevi [20]. However, it has been reported that people in middle Europe are prone to suffer from vitamin D deficiency, especially during the winter months [21]. Because vitamin D levels were not measured in this study, we cannot draw a clear connection between smoking and the prevalence of nevi due to vitamin D deficiency alone. However, we encourage further investigations of this question.

Solar lentigines are typically small, sharply circumscribed, pigmented spots on sun-exposed, skin-aged areas and are considered a sign of photodamage. They are linked to chronic cumulative sun exposure. Bastiaens et al. found that solar lentigines were linked not only to high intermittent sun exposure but also to painful sunburns during childhood and adolescence [22]. We found no evidence that smoking contributes positively or negatively to the prevalence of lentigines, with the exception of the age group from 41 to 50 years, but this result must be interpreted with caution because of the small number of participants.

This study has several limitations. The number of participants was limited, and a multivariate analysis was not possible; an analysis of a larger group might be of interest. Furthermore, the study population may have been drawn from a specific group of people who were interested in health, because the study was performed on volunteers in a public swimming bath during the summer. Moreover, the results could have been affected by inter- and intra-individual differences in the evaluation of melanocytic lesions, despite interobserver training.

## Conclusion

Our results show no correlation between tobacco smoking and the occurrence of nevi, atypical nevi, and lentigines on sun-exposed and non-sun-exposed areas. The well-documented effects of tobacco smoking on the immune system and on cancer development may be exerted through pathways apart from nevogenesis. The biological mechanisms behind these findings are still unclear and remain to be elucidated.

## Supporting information

S1 Data (PDF)
