# Supplementary material for: Prevalence of nevi, atypical nevi, and lentigines in relation to tobacco smoking
Source: PLoS One. 2021 Jul 20;16(7):e0254772. doi: 10.1371/journal.pone.0254772 (PMC8291632; doi:10.1371/journal.pone.0254772)
Supplement: S1 Data — (PDF) [file pone.0254772.s001.pdf]

| sex | age | did at least c | have you be | Did you hav | If yes, was it | Had someor | if yes, relativ |
|---|---|---|---|---|---|---|---|
| 2 | 48 | 1 | 1 | 0 | 0 | 0 | 0 |
| 1 | 52 | 1 | 1 | 0 | 0 | 0 | 0 |
| 2 | 57 | 1 | 2 | 2 | 2 | 2 | 0 |
| 2 | 45 | 1 | 1 | 2 | 0 | 2 | 0 |
| 2 | 41 | 1 | 2 | 2 | 0 | 2 | 0 |
| 1 | 69 | 1 | 1 | 2 | 0 | 2 | 0 |
| 2 | 62 | 1 | 1 | 0 | 0 | 0 | 0 |
| 1 | 37 | 1 | 2 | 2 | 0 | 1 | 2 |
| 1 | 32 | 5 | 1 | 2 | 2 | 1 | 0 |
| 1 | 39 | 1 | 1 | 2 | 2 | 2 | 0 |
| 2 | 32 | 0 | 2 | 2 | 2 | 0 | 0 |
| 1 | 31 | 1 | 1 | 2 | 0 | 2 | 0 |
| 1 | 47 | 0 | 1 | 2 | 0 | 2 | 0 |
| 1 | 42 | 1 | 1 | 2 | 0 | 2 | 0 |
| 2 | 46 | 1 | 1 | 2 | 2 | 2 | 0 |
| 1 | 45 | 1 | 1 | 2 | 0 | 2 | 0 |
| 2 | 49 | 1 | 1 | 2 | 2 | 2 | 0 |
| 2 | 36 | 4 | 1 | 2 | 0 | 2 | 0 |
| 2 | 30 | 1 | 1 | 2 | 0 | 0 | 0 |
| 1 | 41 | 0 | 1 | 2 | 0 | 1 | 1 |
| 1 | 45 | 3 | 2 | 2 | 0 | 2 | 0 |
| 1 | 56 | 1 | 1 | 2 | 0 | 2 | 0 |
| 1 | 55 | 1 | 1 | 2 | 2 | 2 | 0 |
| 1 | 60 | 1 | 1 | 2 | 0 | 2 | 0 |
| 2 | 72 | 1 | 1 | 2 | 0 | 0 | 0 |
| 1 | 62 | 1 | 2 | 1 | 3 | 2 | 0 |
| 1 | 65 | 1 | 2 | 2 | 2 | 2 | 0 |
| 1 | 69 | 1 | 1 | 2 | 0 | 2 | 0 |
| 1 | 62 | 1 | 1 | 2 | 2 | 0 | 1 |
| 1 | 68 | 1 | 1 | 2 | 0 | 2 | 0 |
| 2 | 62 | 1 | 1 | 2 | 0 | 2 | 0 |
| 2 | 58 | 0 | 0 | 0 | 0 | 0 | 0 |
| 1 | 54 | 1 | 1 | 2 | 0 | 2 | 0 |
| 2 | 54 | 1 | 1 | 2 | 2 | 2 | 0 |
| 2 | 55 | 1 | 1 | 2 | 0 | 2 | 0 |
| 1 | 54 | 0 | 0 | 2 | 0 | 2 | 0 |
| 2 | 69 | 5 | 2 | 2 | 2 | 2 | 0 |
| 2 | 65 | 1 | 2 | 0 | 1 | 2 | 0 |
| 2 | 65 | 1 | 1 | 2 | 0 | 2 | 0 |
| 2 | 65 | 1 | 2 | 2 | 0 | 2 | 0 |
| 2 | 69 | 2 | 1 | 2 | 0 | 0 | 0 |
| 2 | 36 | 5 | 2 | 2 | 0 | 1 | 1 |
| 1 | 32 | 0 | 2 | 2 | 3 | 2 | 0 |
| 1 | 37 | 1 | 1 | 2 | 0 | 2 | 0 |
| 2 | 40 | 1 | 2 | 2 | 0 | 2 | 0 |
| 1 | 34 | 3 | 2 | 2 | 0 | 2 | 0 |
| 2 | 42 | 1 | 2 | 2 | 2 | 2 | 0 |
| 2 | 43 | 1 | 1 | 2 | 0 | 2 | 0 |
| 2 | 48 | 1 | 2 | 2 | 2 | 2 | 0 |
| 1 | 45 | 0 | 1 | 2 | 0 | 2 | 0 |
| 1 | 33 | 3 | 2 | 2 | 0 | 2 | 0 |

| | | | | | | | |
|---|---|---|---|---|---|---|---|
| 2 | 31 | 1 | 1 | 2 | 0 | 2 | 0 |
| 1 | 31 | 3 | 1 | 2 | 0 | 0 | 0 |
| 2 | 38 | 1 | 1 | 2 | 0 | 1 | 0 |
| 1 | 39 | 6 | 0 | 2 | 0 | 2 | 0 |
| 1 | 43 | 1 | 2 | 2 | 2 | 2 | 0 |
| 2 | 44 | 1 | 0 | 2 | 0 | 2 | 0 |
| 2 | 42 | 1 | 1 | 2 | 0 | 2 | 0 |
| 2 | 48 | 1 | 1 | 2 | 0 | 2 | 0 |
| 1 | 45 | 1 | 2 | 2 | 0 | 2 | 0 |
| 1 | 41 | 1 | 2 | 2 | 0 | 2 | 0 |
| 1 | 52 | 1 | 1 | 2 | 0 | 0 | 0 |
| 1 | 58 | 5 | 2 | 2 | 2 | 0 | 0 |
| 2 | 60 | 1 | 1 | 0 | 0 | 2 | 0 |
| 2 | 59 | 1 | 2 | 2 | 1 | 2 | 0 |
| 1 | 61 | 0 | 2 | 0 | 2 | 0 | 0 |
| 1 | 68 | 1 | 2 | 1 | 2 | 0 | 1 |
| 2 | 69 | 1 | 2 | 1 | 0 | 0 | 0 |
| 2 | 69 | 6 | 1 | 2 | 0 | 2 | 0 |
| 2 | 59 | 1 | 1 | 2 | 0 | 2 | 0 |
| 2 | 58 | 1 | 1 | 2 | 0 | 2 | 0 |
| 2 | 61 | 1 | 2 | 2 | 0 | 2 | 0 |
| 2 | 71 | 1 | 2 | 2 | 0 | 2 | 0 |
| 1 | 38 | 1 | 2 | 2 | 0 | 2 | 0 |
| 2 | 37 | 1 | 1 | 2 | 3 | 2 | 0 |
| 1 | 43 | 1 | 1 | 2 | 0 | 2 | 0 |
| 2 | 41 | 1 | 2 | 2 | 0 | 2 | 0 |
| 1 | 43 | 6 | 2 | 2 | 2 | 2 | 0 |
| 2 | 46 | 1 | 1 | 2 | 0 | 2 | 0 |
| 2 | 37 | 1 | 1 | 2 | 2 | 2 | 0 |
| 1 | 38 | 1 | 1 | 2 | 2 | 2 | 0 |
| 2 | 39 | 1 | 2 | 2 | 0 | 2 | 0 |
| 1 | 44 | 1 | 2 | 2 | 0 | 1 | 0 |
| 1 | 49 | 1 | 2 | 2 | 0 | 2 | 0 |
| 1 | 48 | 5 | 1 | 2 | 0 | 1 | 1 |
| 2 | 50 | 2 | 2 | 2 | 0 | 2 | 0 |
| 2 | 46 | 1 | 2 | 2 | 0 | 2 | 0 |
| 2 | 43 | 0 | 2 | 2 | 2 | 2 | 0 |
| 2 | 49 | 1 | 2 | 2 | 0 | 1 | 1 |
| 1 | 47 | 1 | 2 | 2 | 0 | 2 | 0 |
| 2 | 43 | 1 | 2 | 2 | 0 | 2 | 0 |
| 2 | 47 | 1 | 1 | 2 | 0 | 2 | 0 |
| 1 | 57 | 5 | 2 | 2 | 2 | 2 | 0 |
| 2 | 63 | 4 | 2 | 0 | 0 | 0 | 0 |
| 1 | 52 | 5 | 2 | 2 | 0 | 2 | 0 |
| 2 | 52 | 1 | 2 | 2 | 2 | 2 | 0 |
| 2 | 60 | 2 | 2 | 2 | 0 | 1 | 1 |
| 2 | 61 | 1 | 1 | 0 | 3 | 0 | 0 |
| 2 | 68 | 1 | 1 | 0 | 0 | 2 | 0 |
| 2 | 78 | 6 | 2 | 1 | 1 | 2 | 0 |
| 1 | 33 | 3 | 2 | 2 | 0 | 2 | 0 |
| 2 | 38 | 1 | 1 | 2 | 0 | 2 | 0 |
| 2 | 37 | 0 | 1 | 2 | 0 | 2 | 0 |

| | | | | | | | |
|---|---|---|---|---|---|---|---|
| 2 | 46 | 1 | 2 | 0 | 2 | 2 | 0 |
| 2 | 49 | 1 | 1 | 2 | 0 | 2 | 0 |
| 1 | 46 | 1 | 2 | 2 | 0 | 0 | 1 |
| 1 | 40 | 5 | 2 | 2 | 0 | 2 | 0 |
| 2 | 36 | 1 | 1 | 2 | 2 | 2 | 0 |
| 2 | 40 | 1 | 1 | 0 | 3 | 2 | 0 |
| 2 | 42 | 3 | 1 | 2 | 0 | 0 | 0 |
| 2 | 45 | 1 | 1 | 0 | 0 | 2 | 0 |
| 1 | 43 | 6 | 0 | 2 | 0 | 2 | 0 |
| 1 | 54 | 1 | 2 | 0 | 0 | 1 | 1 |
| 2 | 70 | 1 | 2 | 2 | 0 | 0 | 0 |
| 2 | 63 | 1 | 1 | 2 | 0 | 0 | 0 |
| 1 | 54 | 3 | 1 | 2 | 0 | 2 | 0 |
| 2 | 61 | 1 | 1 | 2 | 0 | 2 | 0 |
| 2 | 69 | 0 | 0 | 0 | 0 | 0 | 0 |
| 2 | 66 | 6 | 2 | 1 | 2 | 0 | 0 |

| If yes, was it | How many s | Had you sur | Did you hav | smoker (1) r | former smo | former smo | former smo |
|---|---|---|---|---|---|---|---|
| 0 | 0 | 0 | 0 | 1 | 0 | 0 | 0 |
| 0 | 0 | 0 | 0 | 1 | 0 | 0 | 0 |
| 2 | 0 | 2 | 2 | 2 | 15 | 15 | 20 |
| 0 | 1 | 1 | 1 | 1 | 0 | 0 | 0 |
| 0 | 1 | 1 | 1 | 1 | 0 | 0 | 0 |
| 0 | 1 | 1 | 1 | 1 | 0 | 0 | 0 |
| 2 | 1 | 1 | 1 | 1 | 0 | 0 | 0 |
| 1 | 2 | 2 | 2 | 1 | 0 | 0 | 0 |
| 0 | 2 | 1 | 4 | 1 | 0 | 0 | 0 |
| 0 | 2 | 1 | 1 | 1 | 0 | 0 | 0 |
| 2 | 2 | 1 | 2 | 1 | 0 | 0 | 0 |
| 0 | 2 | 1 | 4 | 1 | 0 | 0 | 0 |
| 0 | 2 | 1 | 2 | 1 | 0 | 0 | 0 |
| 0 | 2 | 1 | 1 | 1 | 0 | 0 | 0 |
| 2 | 2 | 1 | 1 | 1 | 0 | 0 | 0 |
| 0 | 2 | 1 | 1 | 1 | 0 | 0 | 0 |
| 0 | 2 | 1 | 2 | 1 | 0 | 0 | 0 |
| 0 | 2 | 1 | 2 | 0 | 0 | 0 | 0 |
| 2 | 2 | 1 | 2 | 0 | 0 | 0 | 0 |
| 0 | 2 | 1 | 4 | 2 | 15 | 20 | 20 |
| 0 | 2 | 1 | 1 | 0 | 0 | 0 | 0 |
| 0 | 2 | 2 | 0 | 1 | 0 | 0 | 0 |
| 0 | 2 | 1 | 2 | 1 | 0 | 0 | 0 |
| 0 | 2 | 1 | 1 | 1 | 0 | 0 | 0 |
| 2 | 2 | 1 | 2 | 1 | 0 | 0 | 0 |
| 0 | 2 | 2 | 2 | 1 | 0 | 0 | 0 |
| 0 | 2 | 1 | 2 | 1 | 0 | 0 | 0 |
| 0 | 2 | 1 | 1 | 1 | 0 | 0 | 0 |
| 1 | 2 | 1 | 2 | 1 | 0 | 0 | 0 |
| 0 | 2 | 1 | 4 | 1 | 0 | 0 | 0 |
| 0 | 2 | 1 | 4 | 1 | 0 | 0 | 0 |
| 0 | 2 | 1 | 2 | 2 | 18 | 33 | 20 |
| 0 | 2 | 2 | 2 | 2 | 15 | 15 | 20 |
| 0 | 2 | 1 | 2 | 0 | 0 | 0 | 0 |
| 0 | 2 | 1 | 2 | 2 | 18 | 31 | 30 |
| 0 | 2 | 2 | 2 | 0 | 0 | 0 | 0 |
| 2 | 2 | 1 | 4 | 2 | 18 | 25 | 20 |
| 0 | 2 | 1 | 2 | 2 | 15 | 45 | 20 |
| 0 | 2 | 2 | 0 | 2 | 18 | 17 | 20 |
| 0 | 2 | 0 | 3 | 2 | 16 | 30 | 30 |
| 0 | 2 | 2 | 1 | 2 | 18 | 35 | 30 |
| 1 | 3 | 1 | 2 | 1 | 0 | 0 | 0 |
| 0 | 3 | 2 | 2 | 1 | 0 | 0 | 0 |
| 0 | 3 | 3 | 4 | 1 | 0 | 0 | 0 |
| 0 | 3 | 1 | 4 | 1 | 0 | 0 | 0 |
| 0 | 3 | 0 | 2 | 1 | 0 | 0 | 0 |
| 0 | 3 | 2 | 2 | 1 | 0 | 0 | 0 |
| 0 | 3 | 1 | 2 | 1 | 0 | 0 | 0 |
| 0 | 3 | 1 | 1 | 1 | 0 | 0 | 0 |
| 0 | 3 | 1 | 4 | 1 | 0 | 0 | 0 |
| 0 | 3 | 1 | 1 | 0 | 0 | 0 | 0 |

| | | | | | | | |
|---|---|---|---|---|---|---|---|
| 0 | 3 | 1 | 2 | 0 | 0 | 0 | 0 |
| 0 | 3 | 2 | 3 | 2 | 15 | 17 | 30 |
| 0 | 3 | 2 | 2 | 2 | 16 | 17 | 30 |
| 0 | 3 | 1 | 2 | 2 | 17 | 15 | 30 |
| 0 | 3 | 1 | 2 | 2 | 12 | 26 | 40 |
| 0 | 3 | 1 | 2 | 2 | 16 | 19 | 20 |
| 0 | 3 | 2 | 4 | 2 | 15 | 20 | 20 |
| 0 | 3 | 1 | 4 | 2 | 15 | 33 | 20 |
| 0 | 3 | 1 | 2 | 0 | 0 | 0 | 0 |
| 0 | 3 | 1 | 2 | 0 | 0 | 0 | 0 |
| 2 | 3 | 2 | 2 | 1 | 0 | 0 | 0 |
| 0 | 3 | 2 | 2 | 1 | 0 | 0 | 0 |
| 0 | 3 | 2 | 2 | 1 | 0 | 0 | 0 |
| 0 | 3 | 2 | 1 | 1 | 0 | 0 | 0 |
| 2 | 3 | 0 | 3 | 1 | 0 | 0 | 0 |
| 1 | 3 | 2 | 1 | 1 | 0 | 0 | 0 |
| 0 | 3 | 2 | 2 | 1 | 0 | 0 | 0 |
| 0 | 3 | 2 | 0 | 1 | 0 | 0 | 0 |
| 0 | 3 | 1 | 4 | 0 | 0 | 0 | 0 |
| 2 | 3 | 2 | 2 | 2 | 16 | 38 | 40 |
| 2 | 3 | 1 | 2 | 2 | 16 | 24 | 30 |
| 0 | 3 | 2 | 2 | 0 | 0 | 0 | 0 |
| 0 | 4 | 1 | 4 | 1 | 0 | 0 | 0 |
| 0 | 4 | 1 | 2 | 1 | 0 | 0 | 0 |
| 0 | 4 | 3 | 3 | 1 | 0 | 0 | 0 |
| 0 | 4 | 2 | 2 | 1 | 0 | 0 | 0 |
| 0 | 4 | 1 | 2 | 1 | 0 | 0 | 0 |
| 2 | 4 | 1 | 1 | 1 | 0 | 0 | 0 |
| 0 | 4 | 1 | 2 | 2 | 16 | 20 | 25 |
| 0 | 4 | 1 | 2 | 0 | 0 | 0 | 0 |
| 0 | 4 | 1 | 4 | 0 | 0 | 0 | 0 |
| 0 | 4 | 2 | 3 | 2 | 15 | 21 | 30 |
| 0 | 4 | 2 | 2 | 2 | 16 | 28 | 20 |
| 3 | 4 | 1 | 3 | 2 | 14 | 33 | 30 |
| 0 | 4 | 1 | 3 | 2 | 15 | 17 | 30 |
| 0 | 4 | 1 | 1 | 2 | 18 | 20 | 20 |
| 0 | 4 | 2 | 2 | 0 | 0 | 0 | 0 |
| 3 | 4 | 2 | 2 | 2 | 18 | 20 | 20 |
| 0 | 4 | 2 | 4 | 0 | 0 | 0 | 0 |
| 0 | 4 | 1 | 2 | 0 | 0 | 0 | 0 |
| 0 | 4 | 1 | 2 | 0 | 0 | 0 | 0 |
| 0 | 4 | 2 | 2 | 1 | 0 | 0 | 0 |
| 3 | 4 | 0 | 2 | 1 | 0 | 0 | 0 |
| 3 | 4 | 2 | 2 | 2 | 15 | 35 | 20 |
| 2 | 4 | 1 | 1 | 2 | 14 | 20 | 40 |
| 1 | 4 | 2 | 4 | 2 | 14 | 30 | 30 |
| 3 | 4 | 2 | 2 | 2 | 17 | 20 | 20 |
| 0 | 4 | 2 | 4 | 2 | 16 | 19 | 40 |
| 0 | 4 | 2 | 2 | 2 | 14 | 18 | 40 |
| 0 | 5 | 1 | 3 | 1 | 0 | 0 | 0 |
| 0 | 5 | 1 | 2 | 1 | 0 | 0 | 0 |
| 0 | 5 | 1 | 4 | 1 | 0 | 0 | 0 |

| | | | | | | | |
|---|---|---|---|---|---|---|---|
| 0 | 5 | 0 | 4 | 1 | 0 | 0 | 0 |
| 0 | 5 | 1 | 2 | 1 | 0 | 0 | 0 |
| 1 | 5 | 1 | 3 | 1 | 0 | 0 | 0 |
| 0 | 5 | 1 | 2 | 2 | 18 | 35 | 20 |
| 0 | 5 | 1 | 3 | 0 | 0 | 0 | 0 |
| 0 | 5 | 1 | 2 | 0 | 0 | 0 | 0 |
| 2 | 5 | 2 | 2 | 2 | 18 | 20 | 20 |
| 0 | 5 | 5 | 4 | 2 | 15 | 20 | 30 |
| 0 | 5 | 1 | 2 | 2 | 16 | 15 | 30 |
| 1 | 5 | 4 | 3 | 1 | 0 | 0 | 0 |
| 2 | 5 | 2 | 2 | 1 | 0 | 0 | 0 |
| 0 | 5 | 3 | 3 | 1 | 0 | 0 | 0 |
| 0 | 5 | 1 | 2 | 2 | 16 | 36 | 20 |
| 0 | 5 | 2 | 3 | 0 | 0 | 0 | 0 |
| 0 | 5 | 2 | 2 | 2 | 15 | 50 | 30 |
| 3 | 5 | 5 | 3 | 2 | 15 | 21 | 100 |

| smoker: ho | smoker: ho | smoker: hov | skin type 1: | actinic lenti | actinic kera | chronic sun | number of r |
|---|---|---|---|---|---|---|---|
| 0 | 0 | 0 | 3 | 2 | 0 | 0 | 6 |
| 0 | 0 | 0 | 2 | 2 | 2 | 2 | 3 |
| 0 | 0 | 0 | 3 | 3 | 1 | 1 | 3 |
| 0 | 0 | 0 | 2 | 1 | 2 | 2 | 2 |
| 0 | 0 | 0 | 2 | 2 | 2 | 0 | 3 |
| 0 | 0 | 0 | 2 | 2 | 0 | 0 | 4 |
| 0 | 0 | 0 | 3 | 2 | 2 | 1 | 2 |
| 0 | 0 | 0 | 2 | 3 | 2 | 2 | 2 |
| 0 | 0 | 0 | 2 | 1 | 2 | 2 | 4 |
| 0 | 0 | 0 | 2 | 3 | 2 | 0 | 2 |
| 0 | 0 | 0 | 3 | 1 | 2 | 2 | 4 |
| 0 | 0 | 0 | 3 | 1 | 2 | 2 | 4 |
| 0 | 0 | 0 | 2 | 2 | 2 | 0 | 3 |
| 0 | 0 | 0 | 2 | 3 | 2 | 0 | 4 |
| 0 | 0 | 0 | 2 | 1 | 2 | 2 | 2 |
| 0 | 0 | 0 | 3 | 2 | 2 | 2 | 4 |
| 0 | 0 | 0 | 3 | 3 | 2 | 2 | 2 |
| 16 | 17 | 30 | 1 | 2 | 2 | 2 | 6 |
| 14 | 11 | 25 | 2 | 1 | 2 | 2 | 4 |
| 0 | 0 | 0 | 3 | 2 | 2 | 0 | 2 |
| 15 | 30 | 20 | 3 | 3 | 2 | 2 | 3 |
| 0 | 0 | 0 | 1 | 3 | 2 | 2 | 4 |
| 0 | 0 | 0 | 2 | 3 | 1 | 1 | 5 |
| 0 | 0 | 0 | 3 | 3 | 2 | 1 | 4 |
| 0 | 0 | 0 | 1 | 3 | 1 | 1 | 4 |
| 0 | 0 | 0 | 1 | 3 | 2 | 1 | 2 |
| 0 | 0 | 0 | 1 | 3 | 2 | 1 | 4 |
| 0 | 0 | 0 | 1 | 2 | 2 | 2 | 5 |
| 0 | 0 | 0 | 2 | 3 | 2 | 1 | 4 |
| 0 | 0 | 0 | 2 | 3 | 2 | 0 | 3 |
| 0 | 0 | 0 | 2 | 2 | 2 | 2 | 3 |
| 0 | 0 | 0 | 1 | 3 | 2 | 2 | 5 |
| 0 | 0 | 0 | 2 | 2 | 2 | 2 | 1 |
| 16 | 38 | 20 | 2 | 1 | 2 | 2 | 4 |
| 0 | 0 | 0 | 3 | 2 | 2 | 2 | 3 |
| 20 | 34 | 25 | 3 | 3 | 2 | 1 | 2 |
| 0 | 0 | 0 | 0 | 3 | 2 | 1 | 3 |
| 0 | 0 | 0 | 2 | 2 | 2 | 2 | 2 |
| 0 | 0 | 0 | 2 | 1 | 2 | 2 | 2 |
| 0 | 0 | 0 | 2 | 1 | 2 | 2 | 2 |
| 0 | 0 | 0 | 3 | 1 | 2 | 2 | 4 |
| 0 | 0 | 0 | 2 | 2 | 2 | 2 | 4 |
| 0 | 0 | 0 | 2 | 3 | 2 | 1 | 6 |
| 0 | 0 | 0 | 2 | 1 | 2 | 2 | 2 |
| 0 | 0 | 0 | 2 | 1 | 2 | 2 | 2 |
| 0 | 0 | 0 | 3 | 1 | 2 | 2 | 2 |
| 0 | 0 | 0 | 2 | 1 | 2 | 2 | 4 |
| 0 | 0 | 0 | 2 | 1 | 0 | 0 | 4 |
| 0 | 0 | 0 | 2 | 1 | 2 | 2 | 4 |
| 0 | 0 | 0 | 3 | 1 | 0 | 0 | 3 |
| 15 | 15 | 20 | 0 | 0 | 0 | 0 | 0 |

| | | | | | | | |
|---|---|---|---|---|---|---|---|
| 15 | 16 | 25 | 2 | 2 | 2 | 2 | 4 |
| 0 | 0 | 0 | 3 | 1 | 2 | 2 | 2 |
| 0 | 0 | 0 | 3 | 2 | 0 | 0 | 3 |
| 0 | 0 | 0 | 3 | 1 | 2 | 2 | 2 |
| 0 | 0 | 0 | 2 | 1 | 2 | 2 | 6 |
| 0 | 0 | 0 | 2 | 1 | 2 | 2 | 4 |
| 0 | 0 | 0 | 2 | 1 | 2 | 2 | 4 |
| 0 | 0 | 0 | 2 | 2 | 2 | 2 | 4 |
| 15 | 30 | 30 | 2 | 1 | 2 | 2 | 3 |
| 16 | 20 | 20 | 3 | 2 | 2 | 2 | 4 |
| 0 | 0 | 0 | 2 | 3 | 2 | 1 | 5 |
| 0 | 0 | 0 | 2 | 3 | 2 | 1 | 2 |
| 0 | 0 | 0 | 3 | 3 | 1 | 1 | 2 |
| 0 | 0 | 0 | 3 | 2 | 2 | 1 | 4 |
| 0 | 0 | 0 | 1 | 3 | 0 | 0 | 4 |
| 0 | 0 | 0 | 2 | 3 | 1 | 1 | 3 |
| 0 | 0 | 0 | 2 | 1 | 2 | 2 | 2 |
| 0 | 0 | 0 | 3 | 2 | 2 | 1 | 3 |
| 20 | 20 | 20 | 1 | 2 | 2 | 2 | 4 |
| 0 | 0 | 0 | 3 | 1 | 2 | 2 | 3 |
| 0 | 0 | 0 | 3 | 3 | 2 | 1 | 4 |
| 17 | 54 | 20 | 3 | 3 | 2 | 1 | 2 |
| 0 | 0 | 0 | 1 | 1 | 2 | 2 | 2 |
| 0 | 0 | 0 | 3 | 2 | 2 | 2 | 2 |
| 0 | 0 | 0 | 1 | 3 | 2 | 1 | 3 |
| 0 | 0 | 0 | 1 | 2 | 0 | 0 | 2 |
| 0 | 0 | 0 | 2 | 1 | 2 | 2 | 4 |
| 0 | 0 | 0 | 2 | 2 | 2 | 2 | 4 |
| 0 | 0 | 0 | 2 | 3 | 2 | 2 | 3 |
| 16 | 22 | 20 | 3 | 3 | 2 | 2 | 3 |
| 20 | 19 | 20 | 3 | 3 | 2 | 2 | 3 |
| 0 | 0 | 0 | 1 | 3 | 2 | 1 | 2 |
| 0 | 0 | 0 | 1 | 3 | 2 | 1 | 3 |
| 0 | 0 | 0 | 2 | 3 | 2 | 2 | 3 |
| 0 | 0 | 0 | 2 | 0 | 2 | 2 | 3 |
| 0 | 0 | 0 | 2 | 1 | 2 | 2 | 4 |
| 14 | 29 | 20 | 2 | 3 | 0 | 0 | 3 |
| 0 | 0 | 0 | 3 | 3 | 2 | 2 | 4 |
| 18 | 29 | 20 | 3 | 2 | 2 | 2 | 4 |
| 14 | 25 | 20 | 3 | 1 | 2 | 2 | 2 |
| 20 | 27 | 30 | 3 | 1 | 2 | 2 | 2 |
| 0 | 0 | 0 | 3 | 2 | 2 | 2 | 4 |
| 0 | 0 | 0 | 2 | 3 | 1 | 1 | 2 |
| 0 | 0 | 0 | 2 | 3 | 2 | 1 | 4 |
| 0 | 0 | 0 | 2 | 2 | 2 | 1 | 3 |
| 0 | 0 | 0 | 3 | 2 | 2 | 2 | 5 |
| 0 | 0 | 0 | 1 | 3 | 2 | 1 | 2 |
| 0 | 0 | 0 | 2 | 3 | 1 | 1 | 6 |
| 0 | 0 | 0 | 2 | 3 | 2 | 0 | 2 |
| 0 | 0 | 0 | 2 | 1 | 2 | 2 | 3 |
| 0 | 0 | 0 | 2 | 1 | 2 | 2 | 2 |
| 0 | 0 | 0 | 2 | 2 | 2 | 2 | 4 |

| | | | | | | | |
|---|---|---|---|---|---|---|---|
| 0 | 0 | 0 | 2 | 1 | 2 | 2 | 2 |
| 0 | 0 | 0 | 2 | 1 | 2 | 2 | 4 |
| 0 | 0 | 0 | 3 | 2 | 2 | 0 | 3 |
| 0 | 0 | 0 | 2 | 2 | 2 | 2 | 2 |
| 16 | 20 | 25 | 2 | 1 | 2 | 2 | 2 |
| 18 | 22 | 25 | 3 | 3 | 0 | 0 | 0 |
| 0 | 0 | 0 | 2 | 1 | 1 | 1 | 5 |
| 0 | 0 | 0 | 2 | 1 | 2 | 2 | 3 |
| 0 | 0 | 0 | 3 | 2 | 2 | 2 | 2 |
| 0 | 0 | 0 | 2 | 2 | 2 | 2 | 4 |
| 0 | 0 | 0 | 3 | 2 | 2 | 1 | 4 |
| 0 | 0 | 0 | 3 | 3 | 1 | 1 | 2 |
| 0 | 0 | 0 | 2 | 3 | 2 | 0 | 3 |
| 17 | 30 | 25 | 1 | 3 | 2 | 1 | 2 |
| 0 | 0 | 0 | 3 | 3 | 1 | 1 | 2 |
| 0 | 0 | 0 | 3 | 3 | 1 | 1 | 3 |

| number aty | template le | template le | template le | template le | template le | template left buttock nu |
|---|---|---|---|---|---|---|
| 3 | 2 | 1 | 3 | 1 | 1 | 0 |
| 1 | 0 | 0 | 7 | 0 | 0 | 0 |
| 1 | 0 | 0 | 7 | 1 | 0 | 0 |
| 1 | 0 | 0 | 0 | 0 | 0 | 0 |
| 1 | 1 | 0 | 3 | 0 | 0 | 1 |
| 1 | 5 | 0 | 30 | 0 | 0 | 0 |
| 1 | 2 | 0 | 5 | 2 | 0 | 0 |
| 4 | 1 | 0 | 2 | 2 | 0 | 0 |
| 1 | 0 | 0 | 0 | 0 | 0 | 0 |
| 1 | 1 | 0 | 20 | 0 | 0 | 2 |
| 1 | 0 | 0 | 0 | 0 | 0 | 0 |
| 1 | 1 | 0 | 0 | 0 | 0 | 0 |
| 2 | 2 | 0 | 4 | 0 | 0 | 0 |
| 1 | 2 | 0 | 11 | 0 | 0 | 0 |
| 1 | 0 | 0 | 0 | 0 | 0 | 0 |
| 1 | 1 | 0 | 0 | 0 | 0 | 0 |
| 1 | 0 | 0 | 50 | 0 | 0 | 0 |
| 2 | 4 | 0 | 0 | 0 | 0 | 0 |
| 2 | 0 | 0 | 7 | 2 | 0 | 0 |
| 2 | 0 | 0 | 2 | 0 | 0 | 1 |
| 1 | 3 | 0 | 17 | 0 | 0 | 0 |
| 1 | 1 | 0 | 3 | 0 | 0 | 0 |
| 1 | 0 | 0 | 40 | 1 | 0 | 0 |
| 2 | 0 | 0 | 14 | 0 | 0 | 2 |
| 1 | 2 | 0 | 5 | 0 | 0 | 0 |
| 1 | 0 | 0 | 50 | 0 | 0 | 0 |
| 1 | 7 | 0 | 15 | 1 | 0 | 0 |
| 1 | 3 | 0 | 5 | 0 | 0 | 0 |
| 1 | 0 | 0 | 8 | 2 | 0 | 0 |
| 1 | 0 | 0 | 50 | 0 | 0 | 0 |
| 2 | 5 | 0 | 30 | 2 | 0 | 0 |
| 1 | 2 | 0 | 15 | 0 | 0 | 0 |
| 0 | 5 | 0 | 0 | 0 | 0 | 0 |
| 3 | 4 | 0 | 0 | 1 | 0 | 0 |
| 2 | 2 | 0 | 5 | 1 | 0 | 2 |
| 1 | 0 | 0 | 50 | 0 | 0 | 0 |
| 1 | 1 | 0 | 40 | 0 | 0 | 0 |
| 1 | 1 | 0 | 50 | 0 | 0 | 0 |
| 1 | 0 | 0 | 10 | 0 | 0 | 0 |
| 1 | 2 | 0 | 30 | 2 | 0 | 0 |
| 1 | 0 | 0 | 0 | 0 | 0 | 0 |
| 2 | 4 | 0 | 2 | 0 | 0 | 0 |
| 4 | 0 | 0 | 27 | 2 | 0 | 0 |
| 1 | 0 | 0 | 6 | 0 | 0 | 0 |
| 1 | 3 | 0 | 40 | 0 | 0 | 0 |
| 1 | 5 | 0 | 0 | 1 | 0 | 0 |
| 2 | 2 | 0 | 15 | 0 | 0 | 0 |
| 1 | 5 | 0 | 40 | 1 | 0 | 0 |
| 3 | 6 | 0 | 2 | 1 | 0 | 0 |
| 2 | 0 | 0 | 0 | 0 | 0 | 0 |
| 0 | 0 | 0 | 0 | 0 | 0 | 0 |

| | | | | | | |
|---|---|---|---|---|---|---|
| 2 | 3 | 0 | 0 | 0 | 0 | 0 |
| 1 | 1 | 0 | 0 | 0 | 0 | 0 |
| 1 | 0 | 0 | 5 | 2 | 0 | 0 |
| 1 | 0 | 0 | 5 | 0 | 0 | 0 |
| 3 | 0 | 0 | 10 | 0 | 0 | 0 |
| 2 | 4 | 0 | 50 | 0 | 0 | 0 |
| 1 | 3 | 0 | 20 | 0 | 0 | 0 |
| 2 | 2 | 0 | 6 | 0 | 0 | 0 |
| 2 | 5 | 0 | 30 | 4 | 0 | 0 |
| 2 | 4 | 0 | 0 | 2 | 0 | 0 |
| 2 | 2 | 0 | 50 | 0 | 0 | 0 |
| 1 | 0 | 0 | 3 | 0 | 0 | 0 |
| 1 | 0 | 0 | 15 | 0 | 0 | 0 |
| 2 | 1 | 0 | 7 | 0 | 0 | 0 |
| 2 | 2 | 0 | 20 | 1 | 0 | 0 |
| 2 | 0 | 0 | 2 | 0 | 0 | 0 |
| 1 | 1 | 0 | 50 | 0 | 0 | 0 |
| 1 | 0 | 0 | 10 | 2 | 0 | 0 |
| 1 | 1 | 0 | 10 | 0 | 0 | 0 |
| 2 | 0 | 0 | 20 | 0 | 0 | 2 |
| 1 | 5 | 0 | 50 | 10 | 0 | 0 |
| 1 | 0 | 0 | 50 | 0 | 0 | 0 |
| 1 | 0 | 0 | 0 | 0 | 0 | 0 |
| 1 | 0 | 0 | 7 | 0 | 0 | 0 |
| 1 | 2 | 0 | 25 | 0 | 0 | 0 |
| 0 | 0 | 0 | 5 | 0 | 0 | 0 |
| 4 | 4 | 0 | 0 | 0 | 0 | 0 |
| 2 | 3 | 0 | 10 | 0 | 0 | 0 |
| 2 | 0 | 0 | 15 | 0 | 0 | 0 |
| 1 | 0 | 0 | 12 | 0 | 0 | 0 |
| 1 | 0 | 0 | 15 | 1 | 0 | 0 |
| 1 | 0 | 0 | 50 | 0 | 0 | 38 |
| 1 | 5 | 0 | 20 | 2 | 0 | 0 |
| 1 | 3 | 0 | 50 | 0 | 0 | 0 |
| 2 | 0 | 0 | 30 | 0 | 0 | 0 |
| 2 | 1 | 0 | 50 | 4 | 0 | 0 |
| 0 | 0 | 0 | 30 | 0 | 0 | 0 |
| 1 | 2 | 0 | 30 | 0 | 0 | 0 |
| 1 | 0 | 0 | 7 | 0 | 0 | 0 |
| 1 | 2 | 0 | 30 | 0 | 0 | 0 |
| 1 | 0 | 0 | 0 | 0 | 0 | 0 |
| 2 | 8 | 0 | 0 | 0 | 0 | 0 |
| 1 | 0 | 0 | 50 | 0 | 0 | 0 |
| 1 | 0 | 0 | 50 | 0 | 0 | 6 |
| 1 | 2 | 0 | 4 | 0 | 0 | 0 |
| 1 | 7 | 0 | 0 | 5 | 0 | 0 |
| 1 | 0 | 0 | 0 | 0 | 0 | 0 |
| 3 | 5 | 0 | 15 | 3 | 0 | 0 |
| 2 | 0 | 0 | 40 | 0 | 0 | 0 |
| 2 | 3 | 0 | 0 | 0 | 0 | 0 |
| 1 | 0 | 0 | 0 | 0 | 0 | 0 |
| 2 | 0 | 0 | 14 | 0 | 0 | 0 |

| | | | | | | |
|---|---|---|---|---|---|---|
| 1 | 2 | 0 | 30 | 0 | 0 | 0 |
| 2 | 4 | 0 | 10 | 0 | 0 | 0 |
| 2 | 2 | 0 | 5 | 0 | 0 | 2 |
| 1 | 1 | 0 | 0 | 2 | 0 | 0 |
| 1 | 4 | 0 | 0 | 0 | 0 | 0 |
| 0 | 7 | 0 | 0 | 6 | 0 | 0 |
| 3 | 1 | 0 | 20 | 1 | 0 | 0 |
| 1 | 2 | 0 | 0 | 1 | 0 | 0 |
| 1 | 0 | 0 | 0 | 0 | 0 | 0 |
| 1 | 5 | 0 | 40 | 2 | 0 | 0 |
| 2 | 0 | 0 | 5 | 2 | 0 | 0 |
| 1 | 2 | 0 | 50 | 0 | 0 | 25 |
| 2 | 0 | 0 | 30 | 0 | 0 | 0 |
| 1 | 7 | 0 | 20 | 1 | 0 | 10 |
| 1 | 0 | 0 | 15 | 0 | 0 | 0 |
| 1 | 0 | 0 | 20 | 0 | 0 | 0 |

umber lentigines
